# Pt‐Regulated Dynamic Cu(0) Regeneration in CuPt Alloy Enables High‐Efficiency Self‐Driven Formaldehyde Fuel Cells for Potassium Formate Production

**DOI:** 10.1002/advs.77928

**Published:** 2026-09-27

**Authors:** Hanxu Hou, Yan Zhou, Weixiang Tang, Desheng Sui, Siman Tao, Xiaohan Li, Hui Zhang, Jiawei Qiang, Wenle Li, Jun Zhang

**Affiliations:** ^1^ Shandong Key Laboratory of Intelligent Energy Materials School of Materials Science and Engineering China University of Petroleum (East China) Qingdao P. R. China; ^2^ State Key Laboratory of Heavy Oil Processing College of Chemical Engineering China University of Petroleum (East China) Qingdao P. R. China; ^3^ College of Chemistry Beijing Normal University Beijing P. R. China

**Keywords:** direct formaldehyde fuel cells, electrocatalysis, electronic modulation, formaldehyde oxidation

## Abstract

Efficient and clean conversion of carbon‐based molecular fuels into electricity without CO_2_ emission remains a great challenge. Herein, formaldehyde was employed as a novel fuel catalyzed by a Cu_30_Pt_X_/CF anode, which was fabricated via electrodeposition. The electronic interaction between Pt and Cu boosts H^*^ dimerization into H_2_ (the rate‐determining step), facilitates the desorption of formic acid, and promotes the regeneration of Cu^0^ active sites, thus enhancing the catalytic activity and stability and broadening the potential window for formaldehyde oxidation. Furthermore, a direct formaldehyde fuel cell was constructed, in which the one‐electron oxidation of formaldehyde proceeds with high selectivity on the Cu anode without the CO_2_ formation pathway. This fuel cell delivers an open‐circuit voltage of 0.94 V and a peak power density of 34.3 mW cm^−2^, and enables the highly selective production of potassium formate while generating electricity spontaneously with a Faradaic efficiency approaching 100%. This work realizes the co‐generation of electrical energy and high‐value‐added chemicals, providing a new strategy and mechanistic basis for the design of high‐performance direct formaldehyde fuel cells (DFFC).

## Graphical abstract

In this work, a Cu_30_Pt_X_/CF anode material was synthesized to effectively catalyze the electrocatalytic formaldehyde oxidation, and thereby assembled a fuel cell that offered a high open‐circuit potential of 0.94 V and a power density of 34.3 mW cm^−2^. This work realizes the co‐generation of electrical energy and high‐value‐added chemicals with nearly 100% Faradaic efficiency.

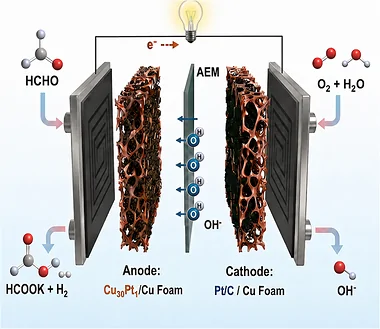

## 1 Introduction

Fuel cells represent efficient, low‐emission devices for direct chemical‐to‐electrical energy conversion [1, 2, 3, 4, 5, 6]. Direct liquid fuel cells based on small organic molecules are especially appealing due to easy fuel handling, compact structure, and wide application. However, conventional fuels, such as methanol, formic acid, and ethanol, face critical drawbacks due to the production of CO_2_ during their oxidation, resulting in greenhouse gas emissions [7, 8, 9, 10, 11, 12]. Moreover, most systems depend on expensive and easily poisoned noble‐metal catalysts [13, 14]. In contrast, formaldehyde (HCHO) stands out as a promising fuel due to its strong reducibility, low thermodynamic oxidation potential (−0.22 V_RHE_), and high volumetric energy density (2.2 kWh L^−1^), which outperforms both methanol and formic acid [15, 16, 17]. It can be obtained from diverse carbon sources, including fossil conversion, biomass, organic waste, and CO_2_ reduction, highlighting its central role in future carbon–energy cycles [17, 18, 19, 20, 21, 22]. More importantly, under appropriate conditions, it can selectively generate value‐added products such as formate and hydrogen, without CO_2_ emission [23, 24, 25, 26]. Notably, potassium formate is a high‐value‐added product with a market price nearly 5 times higher than that of formaldehyde, and is widely used as a deicing agent, reducing agent, and feed additive [27, 28, 29]. When coupled with the oxygen reduction reaction (ORR), formaldehyde oxidation reaction (FOR) can drive spontaneous electricity generation without external energy input [30]. However, the practical development of HCHO fuel cells relies on efficient and stable anode catalysts for FOR.

Cu‐based materials are attractive FOR anodes owing to their low cost and high intrinsic activity for aldehyde oxidation [31, 32, 33]. Cu catalysts can drive FOR at extremely low potentials (down to 0.10 V_RHE_) with nearly 100% Faradaic efficiency toward formate and hydrogen formation [34, 35]. Metallic Cu^0^ serves as the active site for HCHO adsorption, C–H cleavage, and intermediate transformation. The critical issue is that Cu^0^ is readily oxidized to Cu^+^ (∼0.5 V_RHE_) and Cu^2+^ (∼0.7 V_RHE_) under anodic potentials, leading to deactivation [36, 37]. Although oxidized Cu species can be chemically reduced back to Cu^0^ by H_2_C(OH)O^−^ derived from HCHO under alkaline conditions, the electrochemical oxidation rate exceeds the chemical reduction rate at high potentials, resulting in the loss of active Cu^0^. The catalytic state of Cu is therefore governed by a competition between electrochemical oxidation and spontaneous chemical reduction [38]. Broadening the FOR potential window thus requires regulation of the dynamic working‐state evolution of Cu under operating conditions. The central challenge is how to maintain a catalytically favorable Cu^0^ state over a wider potential range, especially under the current densities relevant to direct fuel‐cell operation.

Atomic doping is a promising way to regulate the electronic structure of Cu, resulting in an elevated formation rate of Cu^0^, such as Pt, Ag, Ni, Fe etc. [39, 40, 41]. The atomic doping can provide surface electronic structure regulation of Cu, making it positively or negatively charged, and can thus modify the absorption energy of formaldyde. However, most of the studies focus on how surface charge modulation can benefit the absorption of formaldyde and desorption of the products [42], but rarely focus on how the surface charge regulation can alter the redox‐ability of charged Cu species such as Cu^+^ and Cu^2+^. Nevertheless, this is essential due to the competition between oxidation of Cu and FOR. Therefore, in this work, Pt was incorporated into the Cu lattice to regulate its surface charge and, therefore, modify its redox‐ability. Due to the higher electronegativity of Pt relative to Cu, electrons transfer from Cu to Pt can occur, rendering Cu slightly positively charged (Cu^δ+^) [43]. Such an electron‐deficient structure tailors the electronic structure and d‐band center of Cu sites, which positively shifts the reduction potential of Cu^2+^/Cu^+^. Consequently, Cu^2+^/Cu^+^ can be more readily reduced back to Cu^0^ by HCHO, thus significantly accelerating the autocatalytic reduction rate. The prepared Cu_30_Pt_1_ alloy showed the highest efficiency toward FOR. By coupling anodic FOR with cathodic ORR, the device achieves spontaneous electricity generation together with selective conversion of HCHO to value‐added formate without external energy input. Recently, significant progress has been made in direct formaldehyde fuel cells (DFFCs). Yang et al. [44]. constructed a formaldehyde‐water fuel cell, achieving an open‐circuit voltage of 0.26 V with bipolar hydrogen production. Zhang et al. [30]. developed a Cu_1.2_Pd_0.8_/CC alloy anode for DFFCs, delivering an open‐circuit voltage of 0.9 V and a peak power density of 100.0 mW mg^−1^_Cu_. However, these studies lack an in‐depth understanding of the dynamic evolution and regulation of Cu active sites during the FOR process. In this work, by further coupling FOR with ORR and utilizing the Pt‐induced electronic modulation of Cu in a CuPt alloy, our device achieves an open‐circuit voltage of 0.94 V and a peak power density of 34.3 mW cm^−2^. More importantly, *operando* Raman and infrared spectroscopies show that the enhancement does not simply originate from additional Pt active sites, but from Pt‐induced regulation of Cu working‐state evolution, which stabilizes a sufficient population of active Cu^0^ species under anodic conditions. This work establishes a direct link of interfacial electronic modulation, dynamic Cu‐state regulation, and device‐level output, and provides a mechanistic basis for the design of efficient direct HCHO fuel cells.

## 2 Results and Discussion

### 2.1 Structural Characterizations of Cu_30_Pt_X_/CF

**FIGURE 1:**
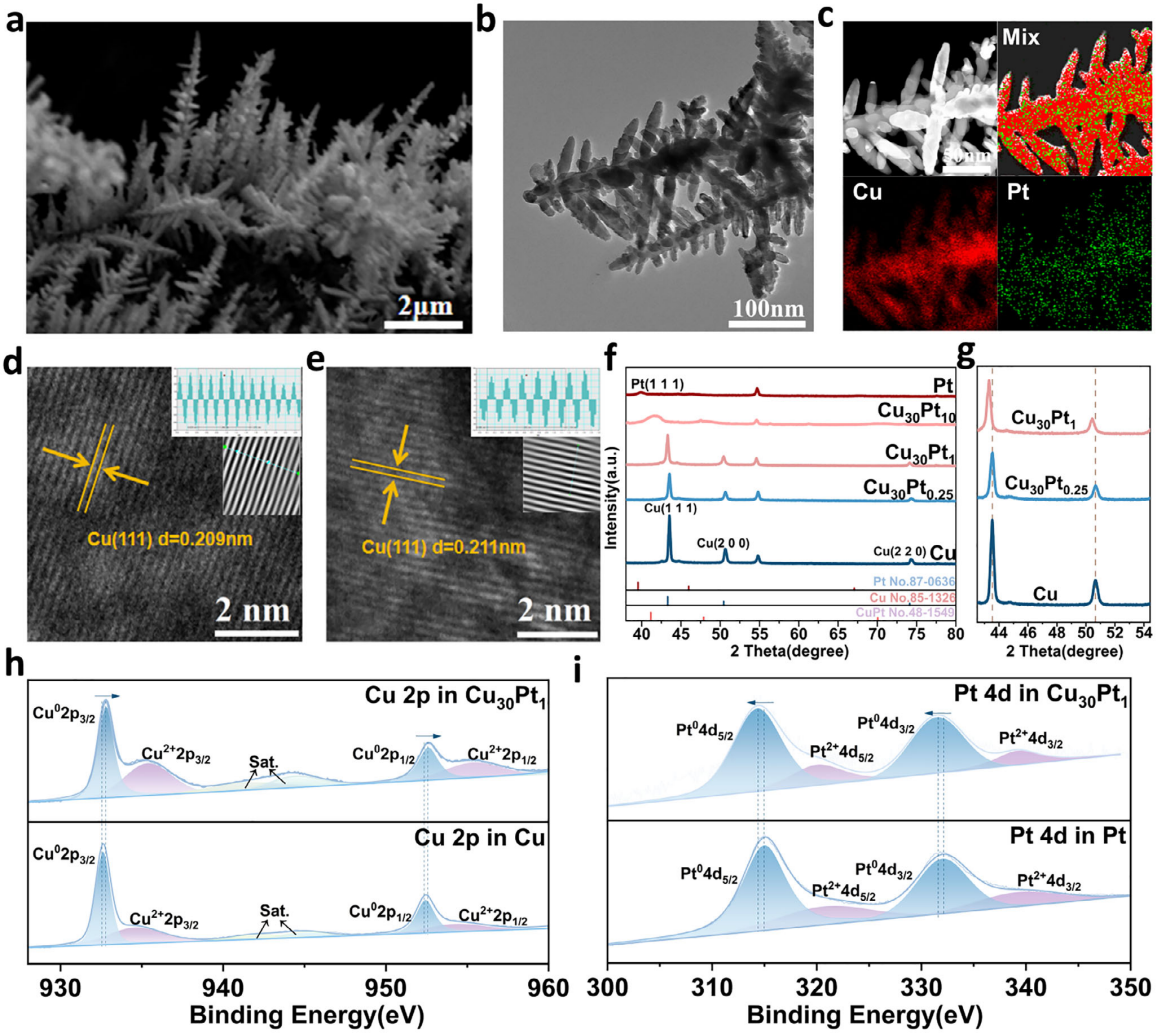
Physical characterization of Cu_30_Pt_1_ catalyst (a) SEM image of Cu_30_Pt_1_/CF. (b) TEM image of Cu_30_Pt_1_. (c) HAADF‐STEM and corresponding elemental mapping images of Cu_30_PtX for Cu and Pt, respectively. HRTEM images of (d) Cu and (e) Cu_30_Pt_1_. (f,g) XRD pattern of Cu_30_Pt_X_/CF. XPS spectra of (h) Cu 2p and (i) Pt 4d.

The Cu_30_Pt_X_/CF catalyst series were prepared on copper foam (CF) by electrodeposition using copper nitrate and chloroplatinic acid as precursors (Figure S1). Scanning electron microscopy (SEM) images show that bare CF has a smooth porous skeleton (Figure S2). After separate electrodeposition of Cu and Pt, Cu/CF exhibits a distinct dendritic structure (Figure S3), while Pt/CF shows a coral‐like morphology (Figure S4). After co‐electrodeposition of both Cu and Pt, Cu_30_Pt_1_/CF uniformly covers the CF surface, and its SEM image reveals a three‐dimensional nano‐dendritic structure (Figure 1a). Transmission electron microscopy (TEM) images further confirm the dendritic morphology of Cu_30_Pt_1_ (Figure 1b). In contrast, Cu/CF consists of nanoparticles with non‐uniform sizes (Figure S5), and Pt/CF forms a dense sheet‐like structure (Figure S6). The energy‐dispersive spectroscopy (EDS) elemental distribution of Cu_30_Pt_1_ in Figure 1c shows that Cu and Pt elements are uniformly distributed without any obvious elemental segregation or enrichment. Moreover, the post‐reaction EDS mapping (Figure S7) confirms that the uniform distribution of Cu and Pt is well retained after the electrocatalytic stability test, indicating the robust structural integrity of the CuPt alloy under operating conditions.

In the high‐resolution transmission electron microscopy (HRTEM) images, the lattice spacing of Cu (111) in Cu_30_Pt_1_ (Figure 1e) is increased by 0.002 nm compared with pure Cu (Figure 1d), indicating a slight lattice expansion after alloying. As shown in the X‐ray diffraction (XRD) patterns (Figure 1f), the peaks of Cu_30_Pt_1_ at 43.61°, 50.61°, and 74.32° correspond to the (111), (200), and (220) crystal planes of Cu (JCPDS No. 85–1326), confirming that Cu_30_Pt_1_ possesses a face‐centered cubic (fcc) crystal structure of Cu. Furthermore, the characteristic diffraction peaks of Cu_30_Pt_1_ shift toward lower angles, which arises from the lattice strain induced by the incorporation of larger‐radius Pt atoms. To quantitatively verify this structural evolution, Rietveld refinement was performed on the XRD data (Figure S8). The refined lattice parameter a increases from 3.615 Å for pure Cu to 3.619 Å for Cu_30_Pt_1_/CF. Moreover, the lattice parameter exhibits a clear linear increase with the Pt atomic fraction (*R* ^2^ = 0.998), confirming the successful alloying of Pt into the Cu lattice. These results agree well with the lattice spacing variation observed by HRTEM, jointly confirming the successful formation of the Cu–Pt alloy. X‐ray photoelectron spectroscopy (XPS) was used to characterize the valence states and changes in the electronic environment of Cu_30_Pt_1_. As shown in the Cu 2p XPS spectra (Figure 1h), the characteristic peaks of Cu^0^ 2p_3/2_ and Cu^0^ 2p_1/2_ are located at 933.5 and 953.5 eV, respectively, while the peaks at 935.1 and 955.4 eV correspond to Cu^2+^ 2p_3/2_ and Cu^2+^ 2p_1/2_. Compared with pure Cu, the binding energy of the Cu 2p orbital in Cu_30_Pt_1_ exhibits a positive shift of approximately 0.2 eV. Figure 1i presents the Pt 4d XPS spectra, where the characteristic peaks of Pt^0^ 4d_5/2_ and Pt^0^ 4d_3/2_ are observed at 314.9 and 332.2 eV, respectively. The two additional binding energy peaks around 321.3 and 339.9 eV are assigned to Pt^2+^ 4d_5/2_ and Pt^2+^ 4d_3/2_. Similarly, the binding energy of the Pt 4d orbital shifts toward lower binding energy by approximately 0.4 eV. The presence of Cu^2+^ and Pt^2+^ characteristic peaks indicates partial oxidation on the surface of the Cu_30_Pt_1_ catalyst, and the content of Cu^2+^ slightly decreases after the reaction (Figures S9 and S10). These results indicate significant charge redistribution and intermetallic electronic interaction in the CuPt alloy, which essentially originates from the electron transfer from Cu to Pt. Thus, the incorporation of Pt into Cu lattice can modulate the electronic structure and dynamic working‐state evolution of Cu during FOR.

### 2.2 Electrochemical Characterizations of Cu_30_Pt_1_/CF and Its Counterparts

**FIGURE 2:**
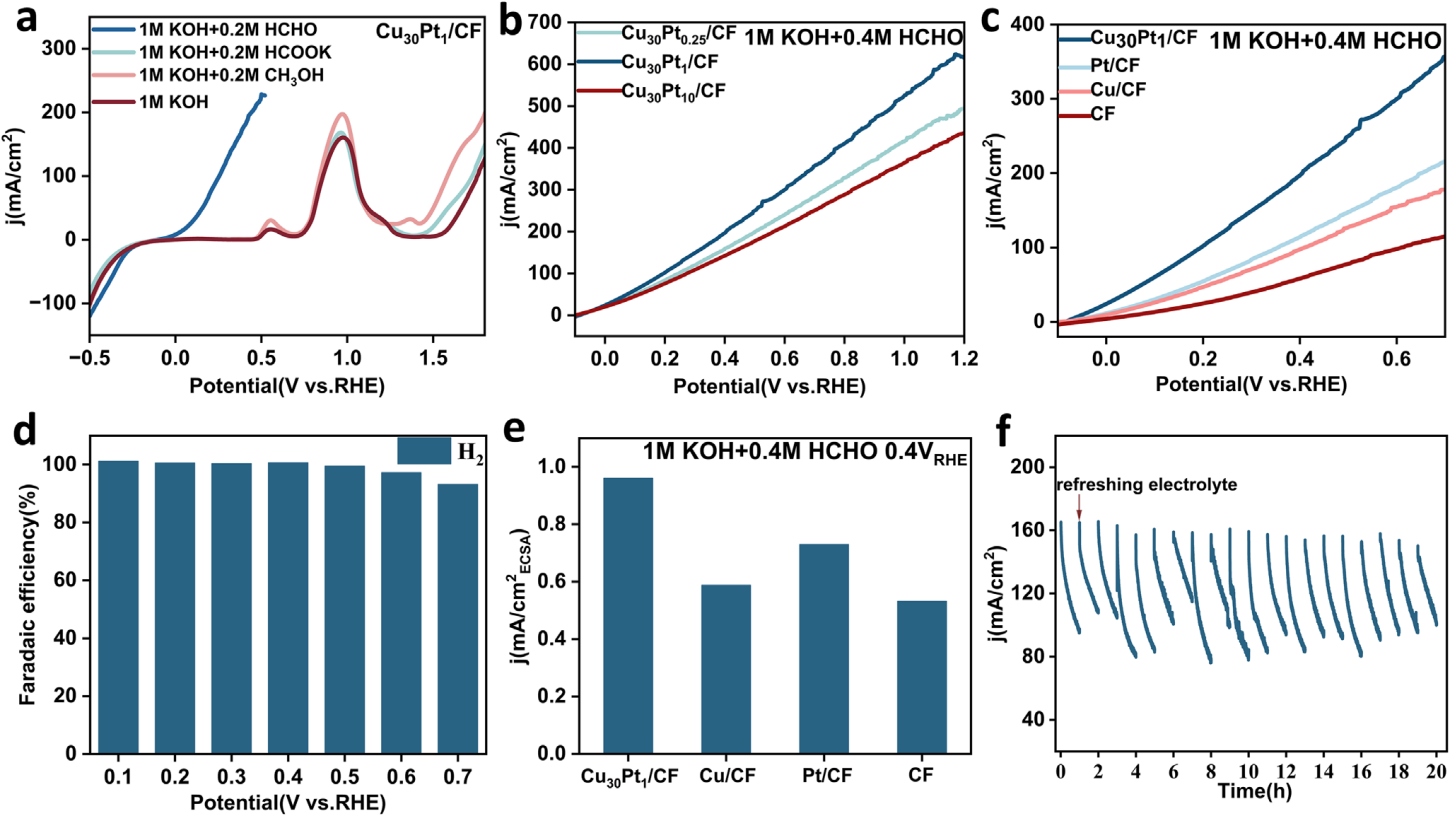
(a) LSV curves of Cu_30_Pt_1_/CF in 1.0 m KOH with different small organic molecules. LSV curves of (b) Cu_30_Pt_0.25_/CF, Cu_30_Pt_1_/CF, Cu_30_Pt_10_/CF (c) Cu_30_Pt_1_/CF, Pt/CF, Cu/CF, CF in 1.0 m KOH + 0.4 m HCHO at a scan rate of 5 mV s^−1^ (without *iR‐correction*). (d) The Faradaic efficiency of Cu_30_Pt_1_/CF at different potentials. (e) ECSA‐normalized current density at 0.4 V_RHE_. (f) The cycling stability of Cu_30_Pt_1_/CF at 0.3 V_RHE_ under 1.0 m KOH 0.4 m HCHO conditions.

It was found that the anodic oxidation of HCHO proceeds via a one‐electron transfer pathway: HCHO first reacts with OH^−^ in the electrolyte to form the (HO)HCHO intermediate adsorbed on the catalyst surface, followed by C─H bond cleavage. The absorbed intermediate H^*^ species then generate H_2_ through the Tafel reaction, and formate (HCOO^−^) as the dominant liquid‐phase product. In order to exclude the interference from methanol (the stabilizer in commercial HCHO solution) and formate oxidation products, controlled experiments by linear sweep voltammetry (LSV) were performed (Figure 2a). Below 1.4 V_RHE_, the oxidation currents of methanol and potassium formate remain at the baseline level, suggesting negligible electrocatalytic activity. In contrast, HCHO displays remarkable oxidation activity accompanied by vigorous gas generation (Figure S11). Subsequently, gas chromatography (GC) was applied to analyze the gaseous products produced at the anode of the H‐type electrolytic cell (Figure S12), confirming that H_2_ is the only gaseous product, with no CO or CO_2_ (the oxidation products of formic acid) detected. To further exclude the possibility that CO_2_ generated from deep oxidation is rapidly absorbed by the alkaline electrolyte in the form of carbonate or bicarbonate, ion chromatography (IC) was employed to analyze the electrolyte before and after electrolysis (Figure S13). The IC results show no detectable increase in carbonate species after the reaction, while formate is identified as the sole liquid‐phase product. These results demonstrate that Cu_30_Pt_1_/CF can efficiently catalyze FOR, while showing no obvious electrocatalytic activity toward the oxidation of formic acid or methanol within the investigated potential range.

The Cu/Pt atomic ratio was regulated by varying the concentration of H_2_PtCl_6_ in the precursor solution. As shown in the linear sweep voltammetry (LSV) curves (Figure 2b and Figure S14), Cu_30_Pt_1_/CF exhibits the best catalytic performance. As presented in Figure 2c, Cu_30_Pt_1_/CF delivers a current density of 200 mA cm^−2^ at 0.4 V_RHE_, which is twice that of Cu/CF and 1.78 times that of Pt/CF. This distinct difference demonstrates that the CuPt alloy regulated with dilute yet electronically coupled Pt is a key factor governing the reaction kinetics of FOR. Figure 2d displays the Faradaic efficiency (FE) of H_2_ for Cu_30_Pt_1_/CF at various potentials. When the potential is below 0.5 V_RHE_, the faradaic oxidation kinetics dominate, and the FE remains nearly 100%. The slight decrease in H_2_ FE beyond 0.5 V_RHE_ is attributed to the partial anodic current contributed by the oxidation of Cu^0^ to Cu^+^.

To further explore the electrochemically active surface areas exposed in the electrolyte, cyclic voltammetry (CV) curves at different scan rates in the non‐faradaic region were recorded for CF, Cu/CF, Pt/CF, and Cu_30_Pt_1_/CF (Figures S15 and S16). The calculated double‐layer capacitances are 10.9, 16.5, 13.7, and 19.1 mF cm^−2^, respectively, indicating that Cu_30_Pt_1_/CF possesses highly exposed active sites. Figure 2e shows the electrochemical surface area (ECSA)‐normalized current densities of CF, Cu/CF, Pt/CF, and Cu_30_Pt_1_/CF at 0.4 V_RHE_. The normalized current density of Cu_30_Pt_1_/CF is significantly higher than those of Pt/CF, Cu/CF, and CF, confirming its superior intrinsic activity. The turnover frequency (TOF) of Cu_30_Pt_1_/CF is calculated to be 1.45 s^−1^, which is 1.7, 1.2, and 1.9 times higher than those of Cu/CF, Pt/CF, and CF, respectively (Figure S17), further evidencing the superior intrinsic catalytic activity of the CuPt alloy. In addition, as shown in Figure S18, the charge transfer resistances (*R*ct) of Cu_30_Pt_1_/CF and Cu/CF are calculated to be 6 and 43 Ω, respectively, revealing faster charge transfer kinetics and better catalytic activity of the former during electrolysis. Consistent with this, the Tafel slope of Cu_30_Pt_1_/CF derived from the LSV curves is significantly lower than those of Cu/CF and Pt/CF (Figure S19), further confirming its superior reaction kinetics for formaldehyde oxidation. Finally, chronoamperometric (i–t) measurements were performed at 0.3 V_RHE_ (Figure 2f). The current density decreases due to the consumption of HCHO, but can recover to near the initial value after refreshing the electrolyte every 1 h, indicating its high catalytic stability.

### 2.3 The Investigation of Electrocatalytic Mechanism of Cu_30_Pt_X_ Alloy

**FIGURE 3:**
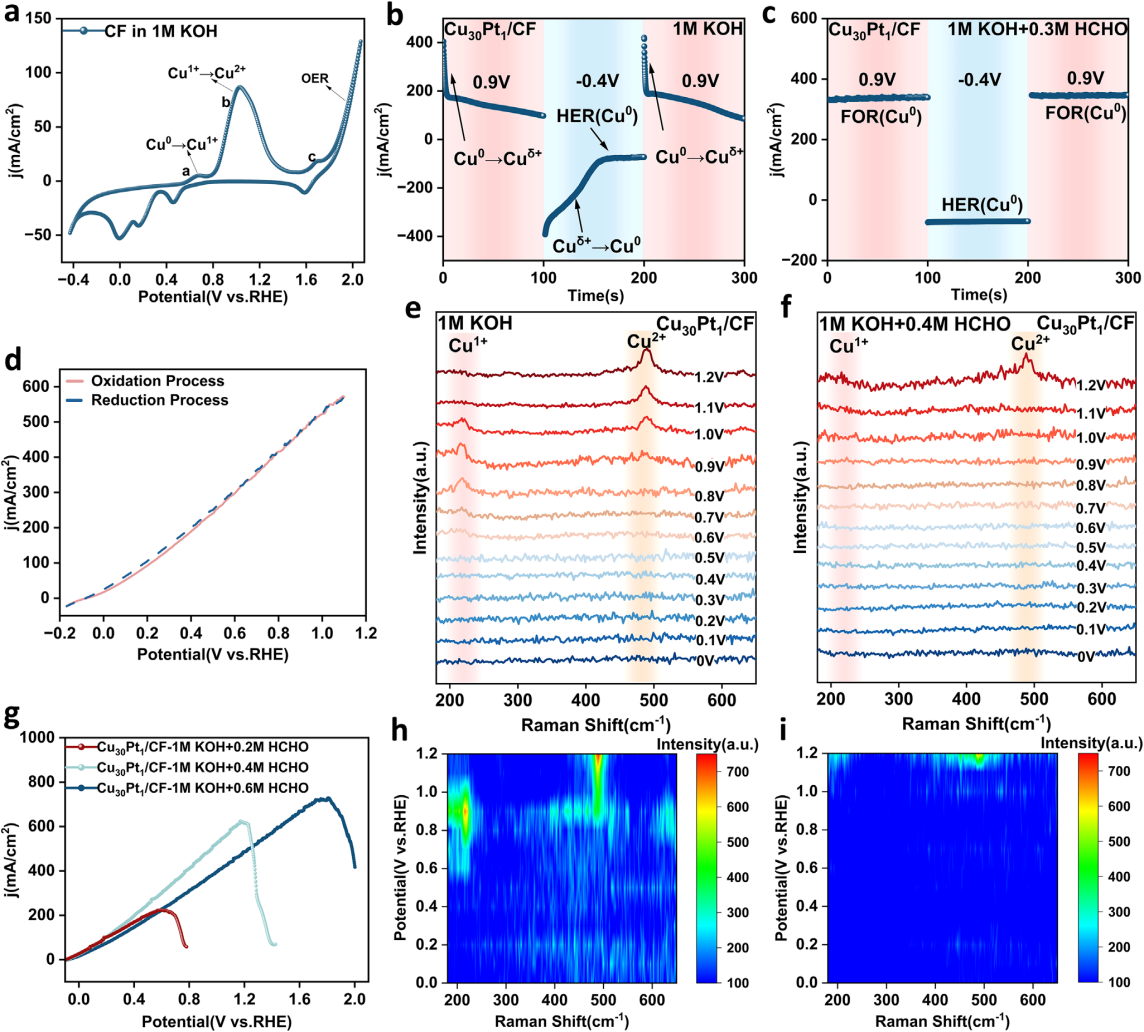
(a) CV curve of the Cu foam (CF) in 1.0 m KOH at a scan rate of 10 mV s^−1^. The periodic chronoamperometry curves of the Cu_30_Pt_1_/CF at 0.9 V_RHE_ and −0.3 V_RHE_ in (b) 1.0 m KOH and (c) 1.0 m KOH + 0.3 m HCHO. (d) CV curves of Cu_30_Pt_1_/CF in 1.0 m KOH + 0.2 m and 0.4 m HCHO at a scan rate of 10 mV s^−1^. (g) LSV curves of Cu_30_Pt_1_/CF at varying HCHO concentrations. *Operando* Raman spectra of the Cu_30_Pt_1_/CF electrode during the electrochemical reaction in (e,h) 1.0 m KOH and (f,i) 1.0 m KOH + 0.4 m HCHO.

In order to verify the dynamic evolution of Cu in CuPt alloy during electrochemical oxidation, the cyclic voltammetry and *operando* Raman spectroscopic characterizations were carried out. Figure 3a presents the cyclic voltammetry (CV) curve of copper foam in 1.0 m KOH solution. Three oxidation peaks and four reduction peaks are identified during the redox process: oxidation peak a is attributed to the conversion of Cu^0^ to Cu^+^; the intense oxidation peak b corresponds to the oxidation of Cu^0^/Cu^+^ to Cu^2+^; and the weak oxidation peak c is assigned to the transformation of CuO/Cu(OH)_2_ to CuOOH. This indicates that pure Cu readily undergo stepwised oxidation under alkaline conditions, and the evolution from Cu^0^ to high‐valence Cu^+^/Cu^2+^ is the core reason for the decreased activity toward FOR at high electrode potential. Figure 3b shows the chronoamperometric behavior of Cu_30_Pt_1_/CF under periodic potential modulation. At an initial potential of 0.9 V_RHE_, an oxidation current corresponding to the conversion of Cu^0^ to Cu^+^/Cu^2+^ was detected, and the current dropped sharply as Cu^0^ was gradually oxidized and consumed. When the potential was switched to −0.4 V_RHE_, the Cu^+^/Cu^2+^ species formed in the previous step were electro‐reduced back to Cu^0^, and the oxidation current was replaced by a reduction current. After the reduction reaction was completed, the current stabilized at a small negative value, corresponding to the HER. When the potential was restored to 0.9 V_RHE_, the above current variation repeated, demonstrating the reversibility of these processes. In contrast, a stable oxidation current was observed at 0.9 V_RHE_ in 1.0 m KOH + 0.3 m HCHO electrolyte (Figure 3c). This indicates that Cu° catalyzes the FOR and is electrochemically oxidized to Cu^+^/Cu^2+^, while H_2_C(OH)O^−^ can rapidly and spontaneously reduce Cu^+^/Cu^2+^ back to Cu^0^ via a spontaneous chemical reaction. Subsequently, when the potential was switched to −0.4 V_RHE_, only a stable hydrogen evolution current was detected in the system, with no obvious reduction current arising from Cu^+^/Cu^2+^. This result confirms that the spontaneous chemical reduction effect of HCHO allows Cu to remain in the zero‐valent state at 0.9 V_RHE_. Namely, within this potential window, the rate of the spontaneous chemical reduction of copper is faster than its electrochemical oxidation rate.

As shown in Figure 3d, upon the addition of 0.4 m formaldehyde, the forward and reverse CV scans completely overlap and exhibit a sustained linear increase in current density, reaching 570 mA cm^−2^ at 1.1 V_RHE_. This significant change, along with the absence of distinct metal oxidation features, indicates that the formaldehyde electro‐oxidation process is substantially superior to the metal oxidation process on the Cu_30_Pt_1_/CF catalyst. However, when the applied potential exceeds 1.1 V_RHE_, a pronounced hysteresis between the forward and reverse CV scans is observed. The cathodic scan displays well‐defined reduction peaks, which are attributed to the reduction of copper oxides generated during the high‐potential polarization process (Figure S20). Based on these observations, we can conclude that, at a fixed formaldehyde concentration and within a certain potential window, the rate of self‐catalytic reduction of Cu^1+^/Cu^2+^ by formaldehyde is greater than the rate of Cu self‐oxidation, meaning that no accumulation of Cu^1+^/Cu^2+^ species occurs on the catalyst surface. We sincerely hope our response meets with your approval.

Figure 3g displays the LSV tests of Cu_30_Pt_1_/CF at different HCHO concentrations, revealing a competitive rate mechanism between electrochemical oxidation and spontaneous chemical reduction. At 0.2 m HCHO, the oxidation current dropped sharply once the potential reached 0.6 V_RHE_. At this point, the rate of Cu^0^ oxidation to Cu^+^ exceeded that of spontaneous chemical reduction by HCHO, leading to the gradual accumulation of inactive Cu^+^ species on the catalyst surface and subsequent termination of FOR. At 0.4 m HCHO, before the potential reached 1.2 V_RHE_, the reduction rate of H_2_C(OH)O^−^ consistently outpaced the oxidation rate of Cu^0^/Cu^+^ to Cu^2+^, ensuring continuous FOR. However, the Cu oxidation rate accelerated with increasing potential. Beyond 1.2 V_RHE_, the electrochemical oxidation rate surpassed the spontaneous chemical reduction rate, causing Cu^2+^ accumulation and eventual catalyst deactivation. Increasing the HCHO concentration to 0.6 m further accelerated the spontaneous chemical reduction rate, thereby significantly broadening the potential window for catalytic FOR. The *operando* Raman spectroscopy results of Cu_30_Pt_1_/CF in Figure 3e,h,f,i further verify the above conclusions. In 1.0 m KOH electrolyte, Cu^+^ began to form on the catalyst surface at 0.6 V_RHE_, and Cu^2+^ appeared at 0.9 V_RHE_. In 1.0 m KOH + 0.4 m HCHO electrolyte, Cu^2+^ was detected on the catalyst surface only at 1.2 V_RHE_, demonstrating that the rate of spontaneous chemical reduction by HCHO was faster than the auto‐oxidation rate of Cu^0^ before 1.2 V_RHE_. In addition, compared with the *operando* Raman results of Cu/CF (Figure S21), the presence of Pt suppressed the accumulation of Cu^2+^ on the catalyst surface.

### 2.4 Verification of Pt‐Promoted Reduction of Cu^2+^/Cu^+^ by Formaldehyde

**FIGURE 4:**
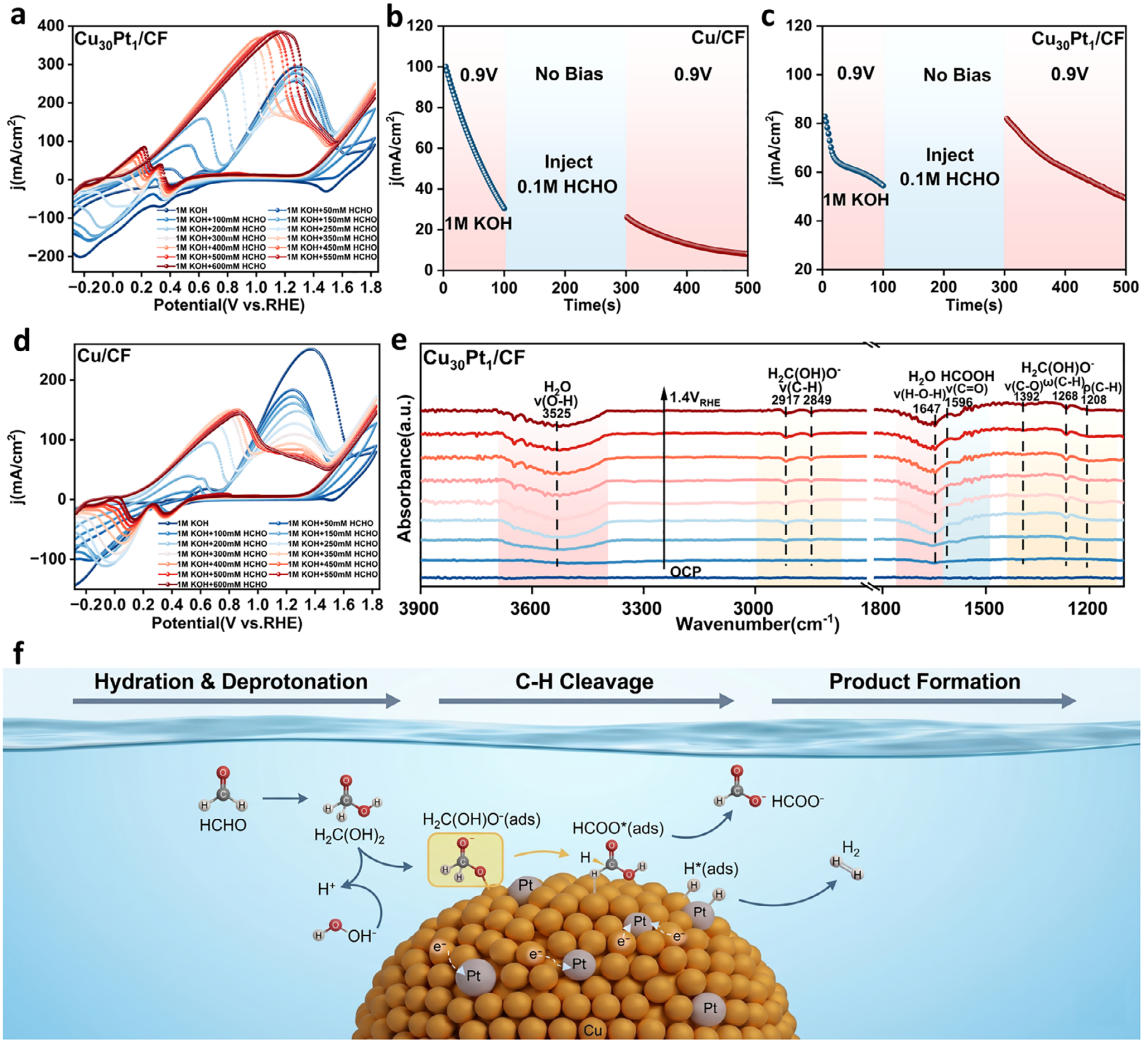
CV curves of (a) Cu_30_Pt_1_/CF and (d) Cu/CF at varying HCHO concentrations. Multistep‐potential chronoamperometry curves of (b) Cu/CF and (c) Cu_30_Pt_1_/CF in 1.0 m KOH at 0.9 V_RHE_ and after injection of 0.1 m HCHO at OCP. (e) *Operando* Fourier transform infrared spectroscopy of the FOR catalyzed by Cu_30_Pt_1_/CF in 1.0 m KOH + 0.4 m HCHO. (f) Schematic illustration of the entire electrocatalytic FOR pathway over the CuPt alloy catalyst.

To directly verify the role of Pt in boosting the spontaneous chemical reduction of HCHO, CV with different concentrations of HCHO were performed (Figure 4a,d). At the same HCHO concentration, the potential window of the FOR on Cu_30_Pt_1_/CF is significantly wider than that on Cu/CF. As the HCHO concentration in the electrolyte increases, the peak area of the reduction peak corresponding to Cu^2+^/Cu^+^ gradually decreases, indicating that HCHO reduces a portion of Cu^2+^/Cu^+^ during the measurement. Moreover, when the HCHO concentration reaches 0.4 m, an obvious FOR current appears near 0.3 V_RHE_ during the reduction process on Cu_30_Pt_1_/CF, whereas this feature is absent on Cu/CF. This observation suggests that Cu^0^ is more readily regenerated on the surface of Cu_30_Pt_1_/CF and continues to catalyze HCHO. Furthermore, once the HCHO concentration exceeds 0.2 m, the catalyst no longer suffers from FOR termination caused by the accumulation of Cu^+^. This implies that Cu^+^ can be more easily reduced by HCHO, thereby enabling the synergistic catalysis of FOR with Cu^0^.

The potential‐step chronoamperometric results in Figure 4b,c provide further evidence for the above conclusion. First, the catalyst surface was subjected to initial polarization oxidation at 0.9 V_RHE_ in 1.0 m KOH electrolyte, and then 0.1 m HCHO was added under open‐circuit potential (OCP) conditions. When the potential of 0.9 V_RHE_ was reapplied, a distinct current surge occurred for Cu_30_Pt_1_/CF, while no such phenomenon was observed for Cu/CF. This implies that the introduction of Pt effectively accelerates the reduction rate of Cu^2+^/Cu^+^ to Cu^0^ by HCHO, leading to a faster regeneration rate of surface Cu^0^ on the Cu_30_Pt_1_/CF material. This is mainly attributed to the fact that Pt optimizes the electronic configuration of Cu, thus stabilizing and retaining sufficient Cu^0^ active sites. Meanwhile, Pt acts as an electron buffer site, accepting electrons donated by H_2_C(OH)O^−^ and transferring them to Cu^+^/Cu^2+^, which accelerates the electron‐acquiring reduction of Cu^+^/Cu^2+^ to Cu^0^ (autocatalytic reduction). The reaction intermediates on the Cu_30_Pt_1_/CF surface during the formic acid oxidation reaction (FOR) were analyzed by *operando* Fourier transform infrared spectroscopy (*operando* FTIR) [45, 46, 47]. As shown in Figure 4e, characteristic peaks assigned to H_2_C(OH)O^−^ were observed, including the C–H stretching vibrations (ν(C–H)) at 2917 and 2849 cm^−1^, the C–O stretching vibration (ν(C–O)) at 1392 cm^−1^, the C–H wagging vibration (ω(C–H)) at 1268 cm^−1^, and the C–H twisting vibration (ρ(C–H)) at 1208 cm^−1^. Most importantly, the characteristic peak corresponding to formic acid (HCOOH) (ν(C = O): 1596 cm^−1^) gradually intensifies with increasing potential, confirming the formation of HCOOH during the FOR process. Thus, we can reasonably speculate that the structural evolution of various species during the FOR process is illustrated in Figure 4f.

### 2.5 DFT Calculations for the Formaldehyde Oxidation Process

**FIGURE 5:**
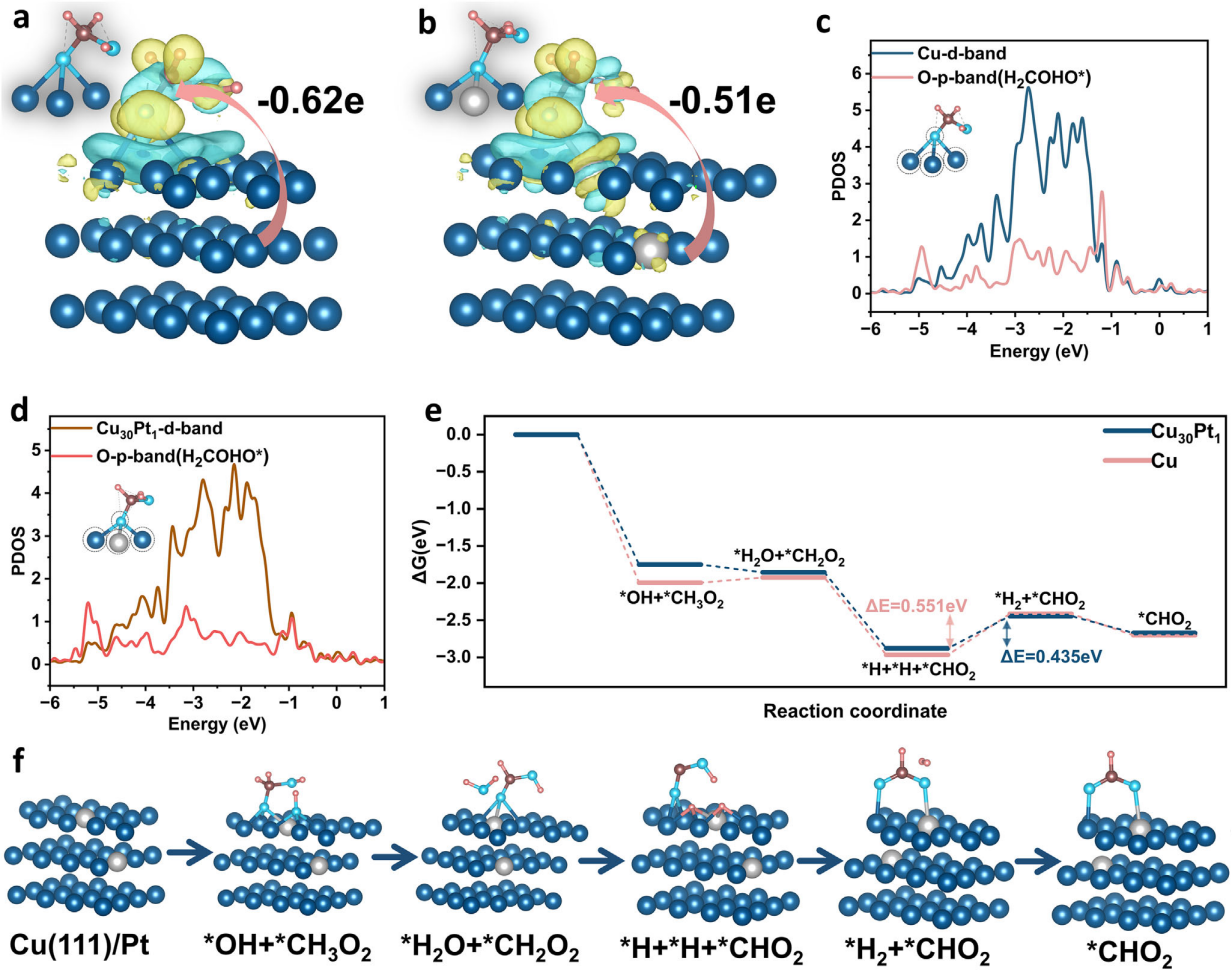
Differential charge density distribution and Bader charge analysis of H_2_C(OH)O^−^ adsorbed on (a) Cu and (b) Cu_30_Pt_1_. Blue and yellow represent electron depletion and accumulation, respectively. The inset illustrations depict the optimized geometries of H_2_C(OH)O^−^ adsorbed on the catalyst surfaces. (c) PDOS of the O atom in H_2_C(OH)O^−^ and the Cu atoms at the adsorption site on Cu. (d) PDOS of the O atom in H_2_C(OH)O^−^ and the Cu and Pt atoms at the adsorption site on Cu_30_Pt_1_. (e) Free‐energy diagram and proposed reaction pathway of the FOR (f) Optimization of crystal structures of different intermediates on Cu(111)_30_Pt_1_.

Theoretical DFT calculations were conducted to examine the structure and energy associated with key steps in the FOR process. The electrostatic potential map (Figure S22) reveals that the C─H bond in H_2_C(OH)O^−^ exhibits a strong polarity, making it prone to cleavage. Structural analysis indicates that both Cu/CF and Cu_30_Pt_1_/CF catalysts predominantly expose the (111) facet (Figure S23), providing a consistent structural basis for the comparative study. Differential charge density results (Figure 5a,b) demonstrate that significant charge transfer occurs upon the adsorption of the H_2_C(OH)O^*^ intermediate on the catalyst surfaces. Bader charge analysis shows that the electron transfer number decreases from 0.62 e on Cu/CF to 0.51 e on Cu_30_Pt_1_/CF. Meanwhile, adsorption energy calculations (Figure S24) show that the adsorption energy of H_2_C(OH)O^−^ on Cu_30_Pt_1_/CF is −1.749 eV, which is higher than −1.9544 eV on Cu/CF, suggesting that the intermediate binds weaker on Cu_30_Pt_1_/CF. This weak adsorption facilitates the rapid desorption of the key product formic acid and avoids the blockage of active sites. Projected density of states (PDOS) analysis (Figure 5c,d) further validates this conclusion: when H_2_C(OH)O^*^ adsorbs on Cu_30_Pt_1_/CF, orbital hybridization occurs between the d‐orbitals of Cu and Pt and the p‐orbital of the O atom in the intermediate, forming resonance peaks, confirming the adsorption interaction. Moreover, the adsorption strength is weaker than that on the pure Cu system, which is highly consistent with the Bader charge analysis. Figure 5f and Figure S25 illustrate the complete reaction pathway of FOR catalyzed by Cu_30_Pt_1_/CF and Cu/CF. In alkaline media, HCHO first forms the H_2_C(OH)O^*^ intermediate, followed by C─H bond cleavage to generate the H_2_COO^*^ intermediate. Further dehydrogenation produces HCOOH^*^ and H^*^, which finally release H_2_ and formate.

Figure 5e compares the Gibbs free energy changes for each step of FOR. On both Cu/CF and Cu_30_Pt_1_/CF, the rate‐determining step is Step 4, the coupling of adsorbed H^*^ to form H_2_, with energy barriers of 0.551 and 0.435 eV, respectively. The remarkable reduction in the energy barrier greatly accelerates the hydrogen dimerization process, which is the primary reason for the superior FOR electrocatalytic performance of the Cu_30_Pt_1_/CF catalyst.

### 2.6 Direct Formaldehyde Fuel Cell Assembly Characterizations With co‐production of Formate

**FIGURE 6:**
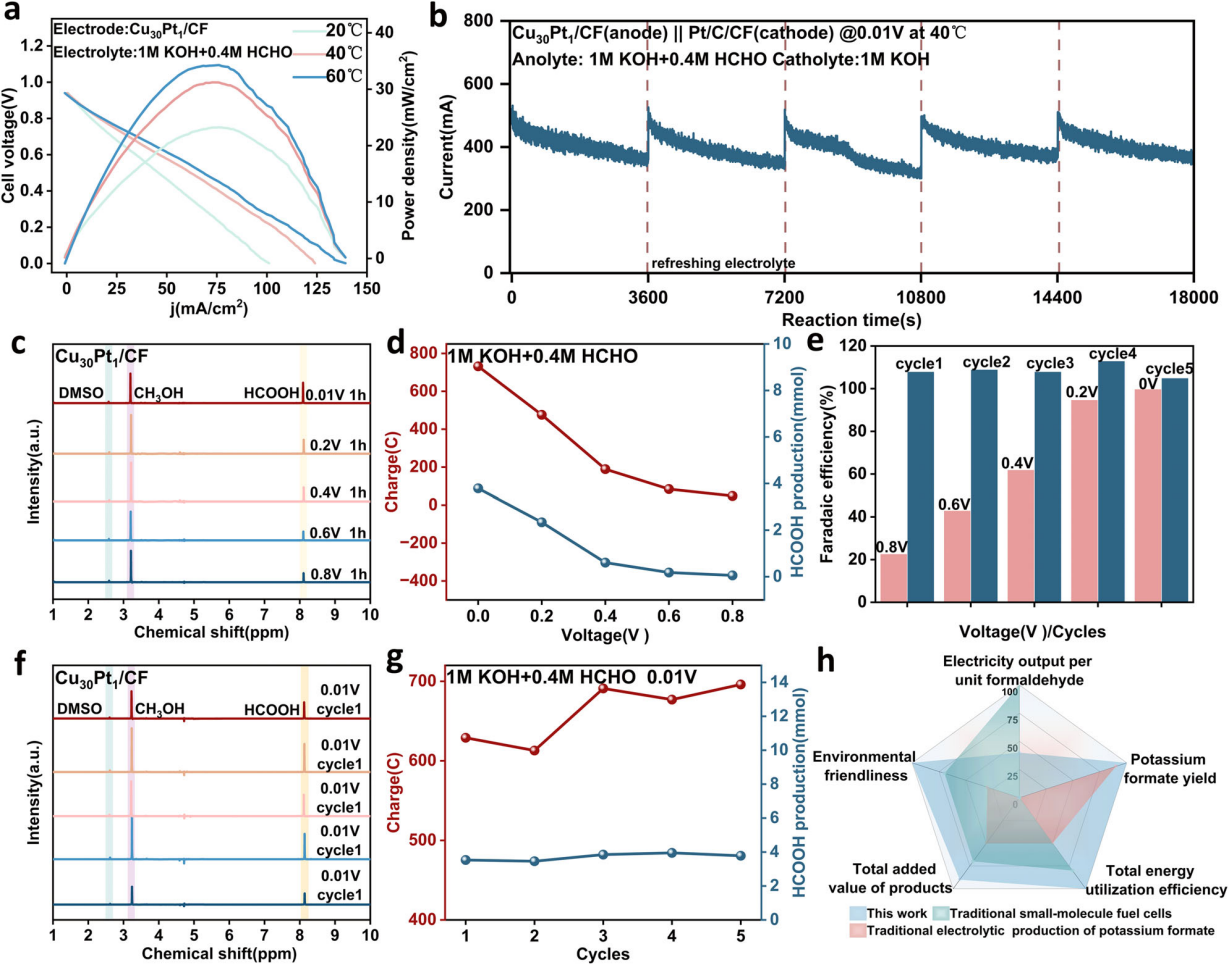
(a) The polarization and power‐density curves for the direct formaldehyde fuel cell. (b) Durability test under a constant cell voltage. (c) ^1^H NMR spectra of the electrolyte for Cu_30_Pt_1_/CF at different potentials; (d) Actual potassium formate production at the anode and corresponding electron utilization efficiency under different cell voltages. (f) ^1^H NMR spectra of the electrolyte for Cu_30_Pt_1_/CF at 0.01 V. (g) Actual potassium formate production at the anode and corresponding electron utilization efficiency at 0.01 V. (e) Bar chart of Faradaic efficiency of potassium formate at different voltages and for five cycles at 0.01 V. (h) Performance Radar Comparison Chart.

In order to show the practical application for DFFC with high‐value‐added products formate and hydrogen gas, a fuel cell assembly was constructed. This fuel cell employs Cu_30_Pt_1_/CF as the anode and Pt/C as the cathode, coupling the FOR with the ORR (Figure S26). It is worth noting that, under the operating conditions, the anode reaction proceeds via a selective partial oxidation pathway, releasing hydrogen gas (HCHO + 2OH^−^ → HCOO^−^ + H_2_ + H_2_O), which has been confirmed by a Faradaic efficiency approaching nearly 100% (Figure 2d). Meanwhile, O_2_ is continuously supplied to the cathode side, where the ORR takes place (O_2_ + 2H_2_O + 4e^−^ → 4OH^−^.) As shown in Figure 6a, the performance of the Cu_30_Pt_1_/CF catalyst at different temperatures was investigated, among which the catalyst exhibited the optimal performance at 60°C, with an open‐circuit voltage (OCV) up to 1 V and a peak power density of 34.3 mW cm^−2^. Subsequently, the stability of the fuel cell was tested at a constant cell voltage of 0.01 V (Figure 6b). Within the initial 1 h of the test, the cell current gradually decreased from 520 to 380 mA, owing to the gradual consumption of HCHO in the electrolyte. The current recovered immediately upon replacement with fresh electrolyte. Additionally, stability testing was performed at 0.5 V (corresponding to the maximum power density, Figure S27). Over a 5‐h testing period, the fuel cell exhibited reproducible performance at different voltages.

Furthermore, the post‐reaction anolyte was collected and characterized by ^1^H nuclear magnetic resonance (^1^H NMR) spectroscopy. As displayed in Figure 6c, only signals corresponding to formic acid and methanol (products of the disproportionation reaction between HCHO and KOH) were observed in the ^1^H NMR spectrum, confirming formic acid as the exclusive product of FOR. The Faradaic efficiencies of potassium formate under different reaction conditions were then calculated based on the standard calibration curves of potassium formate and methanol (Figure S28) and the ^1^H NMR results. As shown in Figure 6d,e, the Faradaic efficiency increased gradually as the cell voltage deviated further from the OCV, reaching 99.8% at short‐circuit potential. This indicates that high‐value‐added potassium formate can still be efficiently produced under practical discharge conditions of the DFFC. Subsequently, five consecutive 1‐h cycle tests were conducted at a cell voltage of the short‐circuit potential to evaluate the stability of formic acid production, verifying that the reaction follows a one‐electron pathway and that the Faradaic efficiency remains stable at approximately 100% in all cycles (Figure 6e–g). The present system achieves the best performance in terms of potassium formate yield, total energy utilization efficiency, total product value‐added, and environmental friendliness, demonstrating comprehensive competitive advantages. Although the electricity output per unit mass of HCHO (0.78 Wh g^−1^) is slightly lower than that of conventional fully oxidized small‐molecule fuel cells (limited by the one‐electron reaction pathway), the practical peak power density (34.3 mW cm^−2^) has reached a considerable level. Moreover, by synchronously producing high‐value‐added potassium formate, the synergistic enhancement of “electricity generation + high value‐added chemical production” is realized, leading to a significantly improved overall economic benefit (Figure 6h). This conversion device can be applied for the reliable and eco‐friendly treatment of HCHO, providing a feasible technical solution for HCHO pollution control and resource utilization.

## 3 Conclusion

In summary, copper‐platinum alloy electrocatalysts with different molar ratios supported on copper foam (Cu_30_Pt_X_/CF) were successfully fabricated and applied to catalyze the anodic formaldehyde oxidation reaction (FOR). Experimental results and density functional theory (DFT) calculations collectively demonstrate that the electronic interaction between Pt and Cu boosts H^*^ dimerization into H_2_ (rate‐determining step), facilitates the desorption of formic acid, and promotes the regeneration of Cu^0^ active sites and thus enhancing the overall electrocatalytic performance, with a current density of up to 100 mA cm^−2^ achieved at 0.2 V_RHE_. Moreover, the electronic coupling between Pt and Cu accelerates the rate of spontaneous chemical reduction of HCHO, namely the regeneration of Cu^0^ active sites, and effectively broadens the potential window for FOR. A DFFC constructed based on the Cu_30_Pt_1_/CF catalyst delivers an open‐circuit voltage of 0.94 V and a peak power density of 34.3 mW cm^−2^. This device enables spontaneous electricity generation while highly selectively producing potassium formate with a Faradaic efficiency close to 100%, thus realizing the co‐generation of electrical energy and high‐value‐added chemicals. This study provides experimental and theoretical support for improving the catalytic mechanism of FOR, and also offers a pivotal catalyst design strategy and technical route for the development of DFFCs with integrated high‐efficiency power generation and high selectivity for value‐added products.

## Author Contributions

**Hanxu Hou**: conceptualization, methodology, investigation, writing – original draft. **Yan Zhou**: conceptualization, validation, funding acquisition, writing – review and editing, supervision. **Weixiang Tang**: data curation. **Desheng Sui**: formal analysis. **Siman Tao**: formal analysis. **Xiaohan Li**: data curation. **Hui Zhang**: formal analysis. **Jiawei Qiang**: methodology, visualization. **Wenle Li**: resources, funding acquisition, validation, project administration. **Jun Zhang**: validation, supervision, funding acquisition, writing – review and editing.

## Conflicts of Interest

The authors declare no conflicts of interest.

## Supporting information




**Supporting File**: advs77928‐sup‐0001‐SuppMat.docx.

## Data Availability

The data that support the findings of this study are available from the corresponding author upon reasonable request.
