# Supplementary material for: Pt‐Regulated Dynamic Cu(0) Regeneration in CuPt Alloy Enables High‐Efficiency Self‐Driven Formaldehyde Fuel Cells for Potassium Formate Production
Source: Adv Sci (Weinh). 2026 Sep 27:e77928. Online ahead of print. doi: 10.1002/advs.77928 (PMC13616309; doi:10.1002/advs.77928)
Supplement: Supplementary file 1 [file ADVS-9999-e77928-s001.docx]

**Supporting Information**

**Pt-Regulated Dynamic Cu(0) Regeneration in CuPt Alloy Enables High-Efficiency Self-Driven Formaldehyde Fuel Cells for Potassium Formate Production**

Hanxu Hou^a^, Yan Zhou^a,*^, Weixiang Tang^a^, Desheng Sui^a^, Siman Tao^a^, Xiaohan Li^a^, Hui Zhang^a^, Jiawei Qiang^c^, Wenle Li^a,*^, Jun Zhang^a,*^

a.Shandong Key Laboratory of Intelligent Energy Materials, School of Materials Science and Engineering	China University of Petroleum (East China)	66 Changjiang West Road, West Coast New Area, ChinaQingdao 266580, P. R. China	E-mail: yanzhou@upc.edu.cn, wli@upc.edu.cn, zhangj@upc.edu.cn

b.State Key Laboratory of Heavy Oil Processing, College of Chemical Engineering China University of Petroleum (East China), 66 Changjiang West Road, West Coast New Area,ChinaQingdao 266580, P. R. China

c.College of Chemistry, Beijing Normal University No.19, Xinjiekouwai Street, Haidian District, Beijing 100875, P. R. China

**Experimental Section**

**Materials.** Chloroplatinic acid (H_2_PtCl_6_, 99%) and copper nitrate (Cu(NO_3_)_2_, 98%) were purchased from Macklin. Formic acid (HCOOH, 99%) was bought from Aladdin. Anhydrous ethanol, sulfuric acid (H_2_SO_4_, 98%) and potassium nitrate (KNO_3_, >99%) were bought from Sinopharm Chemical Reagent Co., Ltd. Copper foam (CF) was purchased from Taobao xingzhenghong scientific corporation. HCl was purchased from Sinopharm Chemical Reagent Co., Ltd. Nafion solution (5%) was purchased from Sigma-Aldrich. The deionized water in the experiment is always ultrapure water (18.5 MΩ·cm^-2^).

**Preparation of Cu_30_Pt_1_/CF.** The integrated Cu_30_Pt_1_/CF was synthesized by the galvanostatic electrodeposition method at ambient temperature. In order to eliminate surface oxides and contamination, CF was ultrasonically treated for 10 min in acetone and 1 M HCl, respectively. The treated CF was cleaned with deionized water and used as the working electrode. Electrodeposition was carried out on a three-electrode system. The reference electrode and counter electrode were a saturated calomel electrode (SCE) and a graphite rod, respectively. The electrolyte is 30 mM Cu(NO_3_)_2_ and 1 mM H_2_PtCl_6_**·**6H_2_O aqueous solution, respectively. During the electrodeposition, CF was treated for 600 s at -0.6 V *vs.* SCE and 600 s at -1.2 V *vs.* SCE, respectively. After electrodeposition, the obtained Cu_30_Pt_1_/CF was distributed on the surface CF. Subsequently, the Cu_30_Pt_1_/CF electrode was cleaned with anhydrous ethanol and deionized water in sequence and dried in a drying oven.

**Preparation of Cu_30_Pt_x_/CF.** All other experimental methods were the same, the only difference being the amount of Cu and Pt. The method was to fix the Cu(NO_3_)_2_ concentration at 30 mM and scale the concentration of H_2_PtCl_6_.

**Characterization.** In order to investigate the morphology and structure of the catalysts, the copper foam was cut and then tested on silicon wafers using a Hitachi S-4800 scanning electron microscope (SEM). Transmission electron microscopy (TEM) and high-resolution TEM (HRTEM) of the catalysts were tested by dropwise addition of the dispersion to a molybdenum mesh using an FEI Tecnai-G2 F30 at an accelerating voltage of 300 kV and the structural features of the catalysts were further characterised. Powder X-ray diffraction (XRD) pattern recording was performed on an X'Pert-PRO MPD diffractometer, which was run with Cu Kα radiation at 40 kV and 40 mA. X-ray photoelectron spectroscopy (XPS) analysis was performed with an Axis Supra spectrometer using a monochromatic Al Kɑ source at 15 mA and 14 kV. Scan analysis with an analysis area of 300 × 700 microns and a pass energy of 100 eV. The spectrum was calibrated by the carbon 1s spectrum, and its main line was set to 284.8 eV, and then the valence state of the catalyst was analyzed using Casa XPS software. The catalyst that has been tested for stability is scraped from the working electrode by ultrasonic treatment and collected for the next step of SEM, TEM and XRD characterization. ^1^H NMR spectra were recorded on a BRUKER AVANCE NEO 500 MHz liquid NMR spectrometer in the indicated solvents. In situ Fourier transform infrared (FTIR) spectrometer (Nicolet IS50, Thermofisher Scientific) was used for infrared spectroscopy.

**Electrochemical measurement**

The electrochemical performance of the samples was evaluated using an electrochemical workstation (Gamry, 5000E) at room temperature. In a typical three-electrode system, the catalyst-loaded copper foam (1 × 1 cm^2^) was directly employed as the working electrode, with a graphite rod serving as the counter electrode and an Hg/HgO electrode as the reference electrode. During the testing process, the positions of the working and reference electrodes were consistently maintained, with the largest surface of the working electrode always facing the reference electrode. The electrolyte used was 1.0 M KOH with or without *x* M HCHO. Experiments requiring the collection of anodic gases were conducted in a two-compartment electrochemical cell separated by an anion exchange membrane (FAA-PK-130, Fumasep). All potentials were referenced to the reversible hydrogen electrode (RHE) using the equation: *E* (vs. RHE) = *E* (*vs.* Hg/ HgO) + 0.059 × pH + 0.098. No *iR* compensation was applied to the LSV and CV curves presented in this study. The linear sweep voltammetry (LSV) curve was measured at a scan rate of 5 mV/s. The electrochemical double-layer capacitance (*C*dl) of the samples was determined using the cyclic voltammetry (CV) method, and *C*dl was calculated using the following equation:

$$C_{dl}=\frac{j_{a}-j_{c}}{2\times v}=\frac{j_{a}+\mid j_{c}\mid}{2\times v}=\frac{\text{Δ}\text{j}}{2\times v}$$

in which *j*_a_ and *j*_c_ are the anodic and cathodic voltammetric current densities, respectively, recorded at the middle of the selected potential range, and v is the scan

rate.

For the direct formaldehyde fuel cell test, 1 mg/cm^2^ Pt/C (40 %, Alfa Aesar) was air-brushed onto 2 × 2 cm^2^ copper foam as a gas diffusion electrode for the oxygen reduction reaction cathode. An anion exchange membrane (3 × 3cm^2^) (FAA-PK-130, Fumasep) was immersed in 1 M KOH for 24h. The membrane should be thoroughly washed with deionized water prior to use to wash off the excess surface KOH. The Cu_30_Pt_X_/CF was directly used as an anode in anodic serpentine flow channels. On the cathode side, a graphitic gas flow channel supplied 100 sccm humidified O_2_ while the anode was circulated with 1 M KOH + 400 mM HCHO electrolyte at a 60 mL min^-1^ flow rate. A scan rate of 5 mV s^-1^ was employed for the LSV test. The cell voltages were recorded without *iR* *correction*.

**ECSA measurements.**

To estimate the ECSA values of the materials, double-layered capacitance (*C*_dl_) was measured using a simple cyclic voltammetry method. *C*_dl_ was further converted into ECSA using the specific capacitance value (~0.1 mF) of a standard 1.0 cm^-2^ surface

$$ECSA=\frac{C_{dl}}{C_{s}}$$

*C*_s_=0.1 mF cm^-2^

**Electrolyte replacement procedure for the durability test**

The detailed procedure for electrolyte replacement in the cyclic durability test (Fig. 2f) is as follows. After each cycle reached 1 hour of testing, the circuit was disconnected to stop the measurement. The anodic electrolyte (containing HCHO and KOH) was then removed, and an equal volume of fresh KOH solution was immediately injected into the cell, followed by the addition of a predetermined amount of HCHO solution. The electrolyte was manually stirred for approximately 10s to ensure homogeneity, after which the circuit was reconnected to initiate the next cycle. The entire replacement process typically took about 20–30s. It should be noted that the electrode was not rinsed at any point during the entire replacement procedure.

**Product Analysis**

The gaseous products were identified using gas chromatography (GC-7920). H_2_ was analyzed with a thermal conductivity detector (TCD). The quantity of H_2_ production was determined using the drainage method. The actual H_2_ produced amount was calculated on the basis of

𝑛 [𝑚𝑜𝑙𝑒] 𝑎𝑐𝑡𝑢𝑎𝑙𝑙𝑦 𝑝𝑟𝑜𝑑𝑢𝑐𝑒𝑑 = 𝑉 / 𝑉𝑚

where *V* is the volume of gas collected, *Vm* (24.5 L/mol) is the molar volume of the gas at 25 C.

The theoretically H_2_ produced amount was calculated on the basis of

𝑛 [𝑚𝑜𝑙𝑒] 𝑡ℎ𝑒𝑜𝑟𝑒𝑡𝑖𝑐𝑎𝑙𝑙𝑦 𝑝𝑟𝑜𝑑𝑢𝑐𝑒𝑑 = 𝑄 / (𝛼 × 𝐹)

where *Q* is the transferred charge, α is the number of electrons transferred for each product molecule (α=2), F is the Faraday constant.

The gas-phase faradaic efficiency (FE) was calculated using the following equation:

𝐹𝐸 (𝑔𝑎𝑠 − 𝑝ℎ𝑎𝑠𝑒) =$\frac{\text{n}[\text{mole}]\text{ actually produced}}{\text{n}[\text{mole}]\text{ theoretically produced}}$× 100%

The liquid phase products were identified and quantified by nuclear magnetic resonance (NMR, Bruker, 600M NEO). To prevent further Cannizzaro reaction, 450 µL of the electrolyte was acidified with 50 µL of 37% HCl. Subsequently, 100 µL of 10 mM DMSO (used as the internal standard) D_2_O solution was added. The ^1^H spectra were obtained using a pre-saturation method to suppress the water peak. The liquid-phase faradaic efficiency (FE) was calculated using the following equation:

𝐹𝐸 (𝑙𝑖𝑞𝑢𝑖𝑑 − 𝑝ℎ𝑎𝑠𝑒) = 𝛼 × 𝑛 × 𝐹 / 𝑄

where α is the number of electrons transferred for the reduction product generation (α=1), n is the total amount of reduction products (in moles), F is the Faraday constant, and Q is the transferred charge.

**Electrochemical In-situ Attenuated Total Reflection Fourier-transform Infrared (ATR−FTIR) Measurements**

Electrochemical ATR−FTIR spectra were recorded using a Fourier transform infrared spectrometer (FTIR, Nicolet iS50) equipped with a liquid-nitrogen-cooled MCT detector. Electrodeposited copper foam was employed as the working electrode. Pt sheet and Ag/AgCl electrodes were used as the counter and reference electrodes, respectively. The potential-dependent ATR−FTIR spectra were recorded from open circuit potential (OCP) to 1.4 V_RHE_ in 1.0 M KOH with 0.2 M HCHO solution by holding the potential for 120 s.

**Electrochemical In-situ Raman Spectroscopy Measurements**

The *Operando* Raman spectra were recorded using a Raman microscope (LabRAM HR Evolution) with a 532 nm laser excitation (power ~17 mW) under controlled potentials, provided by an electrochemical workstation. Specifically, the Cu_30_Pt_1_/CF, Ag/AgCl electrode and a carbon rod were employed as the working, reference, and counter electrodes, respectively. The electrolyte consisted of 1.0 M KOH, with or without 0.4 M HCHO, and was circulated using a peristaltic pump. Raman spectra were collected at various potentials (*E* = 0, 0.1, 0.2, 0.3, 0.4, 0.5, 0.6, 0.7, 0.8, 0.9, 1.0, 1.1, 1.2 V *vs*. RHE) after a 60-s duration at each potential.

**Computational Method**

All density functional theory (DFT) calculations were performed using the plane-wave pseudopotential method as implemented in the CASTEP module in Materials Studio. The exchange–correlation interactions were described by the generalized gradient approximation (GGA) with the Perdew–Burke–Ernzerhof (PBE) functional. Ultrasoft pseudopotentials were used to represent the electron–ion interactions.

Geometry optimizations were carried out using the Broyden–Fletcher–Goldfarb–Shanno (BFGS) algorithm. The convergence criteria were set as follows: the total energy change was less than 1.0 × 10^-5^ eV/atom, the maximum force was below 0.01 eV/Å, the maximum stress was less than 0.02 GPa, and the maximum atomic displacement was within 5.0 × 10^-5^ Å. For slab models, a vacuum layer of 15 Å was introduced along the direction perpendicular to the surface to avoid interactions between periodic images.

The density of states (DOS) was calculated based on the optimized structures. The adsorption energy was calculated to evaluate the interaction between the adsorbate and the substrate. The adsorption energy was defined as the total energy difference between the adsorption system and the sum of the energies of the isolated substrate and adsorbate. All structures involved in adsorption energy calculations were fully optimized using the same computational settings described above. A negative adsorption energy indicates a thermodynamically favorable adsorption process.

The reaction free energy was further calculated to evaluate the catalytic performance. The Gibbs free energy change was obtained by correcting the DFT total energy with zero-point energy (ZPE) and entropy contributions. The free energy change of each elementary step was used to construct the reaction energy profile.

**Figures**

**H_2_PtCl_6_·6H_2_O**

**Cu(NO_3_)_2_**

**electrodeposition**

**Figure S1*.*** The electrodeposition process of Cu_30_Pt_X_ deposited on copper foam *via* constant potential electrodeposition.

**Figure S2.** SEM image of CF.

**2μm**

**Figure S3.** SEM image of Cu/CF

**Figure S4.** SEM image of Pt/CF

**Figure S5.** TEM image of Cu/CF.

**Figure S6.** TEM image of Pt/CF

**Figure S7.** HAADF-STEM image and corresponding EDX maps of Cu_30_Pt_1_/CF after stability test for Cu and Pt, respectively.

**Figure S8.** XRD Rietveld refinement patterns of (a) Cu, (b) Cu_30_Pt_0.25_/CF, and (c) Cu_30_Pt_1_/CF. (d) The linear relationship between the lattice parameter a and the Pt atomic fraction.

**Figure S9.** XPS spectra of Cu/CF before and after reaction at 0.5 V_RHE_.

**Figure S10.** XPS spectra of Cu_30_Pt_1_/CF before and after reaction at 0.5 V_RHE_.

**Figure S11.** Optical image of catalytic FOR in a three-electrode syste

**TCD**

**Signal(mV)**

**H_2_**

**Time(min)**

**TCD**

**Signal(mV)**

**H_2_**

**Time(min)**

**Figure S12.** The gas chromatography (TCD) of gas products collected from an anode chamber in a two-chamber electrolyzer consisting of Cu_30_Pt_1_/CF anode and C rod cathode at an anodic potential of 1.0 V_RHE_, in which 1.0 M KOH was used as the catholyte and 1.0 M KOH with 0.4 M HCHO as the anolyte.

**Figure S13.** Ion chromatograms of the electrolyte before (a) and after (b) electrolysis on Cu_30_Pt_1_/CF at 1.2 V_RHE_ in 1.0 M KOH + 0.4 M HCHO.

**(a)**

**(b)**

**Figure S14.** LSV curves of catalysts with various Cu/Pt ratios in (a) 1.0 M KOH with 0.2 M HCHO and (b) 1.0 M KOH with 0.4 M HCHO.

**Figure S15.** CV curves for the (a) commercial Cu foam, (b) Cu/CF, (c) Pt/CF, and (d) Cu_30_Pt_1_/CF measured with the scan rate ranging from 20 to 100 mV/s.

**Figure S16.** Capacitive current at the middle potential of CV curves as a function of scan rates for Cu_30_Pt_1_/CF, Pt/CF, Cu/CF and CF catalysts.

**Figure S17.** Turnover frequency (TOF) of Cu_30_Pt_1_/CF, Pt/CF, Cu/CF, and CF at 0.4 V_RHE_

**Figure S18.** EIS pattern of Cu30Pt_1_/CF, Pt/CF, Cu/CF and CF in 1.0 M KOH with 0.2 M HCHO.

**Figure S19.** Tafel plots of Cu_30_Pt_1_/CF, Pt/CF, Cu/CF, and CF for FOR in 1.0 M KOH + 0.4 M HCHO.

**Figure S20.** CV curves of Cu_30_Pt_1_/CF in 1.0 M KOH + 0.4 M HCHO at a scan rate of 10 mV s^−1^

**(b)**

**(a)**

**Figure S21.** *Operando* Raman spectra of the Cu/CF electrode during the electrochemical reaction in (a) 1.0 M KOH and (b) 1.0 M KOH with 0.4 M HCHO.

**Figure S22.** Calculated electrostatic potential maps of HCHO, H_2_C(OH)_2_, and H_2_C(OH)O^−^ under vacuum conditions.

Owing to the weak nucleophilicity of water, formaldehyde (HCHO) is sufficiently hydrated and predominantly exists as **H_2_C(OH)_2_** in aqueous solution. Under alkaline conditions, **H_2_C(OH)_2_** undergoes **single deprotonation** by losing a proton from one hydroxyl group to yield the **H_2_C(OH)O**^−^ intermediate. Although a further increase in pH may trigger a second deprotonation to form **H_2_CO_2_^2-^**, this process is thermodynamically and kinetically unfavorable. Accordingly, this work identifies **H_2_C(OH)O**^−^ as the actual reactive intermediate for electrocatalytic formaldehyde oxidation, which is fully supported by our in situ FTIR measurements. To uncover the cleavage tendency of the C–H bond, comparative analysis based on **electrostatic potential distribution** was performed on key intermediates. The C–H bonds in both HCHO and **H_2_C(OH)_2_** show uniform charge distribution and weak polarity, making them difficult to break. In sharp contrast, the C–H bond in **H_2_C(OH)O**^−^ exhibits obvious charge separation and strong polarity, which significantly lowers the energy barrier for bond cleavage. These results clarify the high reactivity of the C–H bond in **H_2_C(OH)O**^−^ at the electronic structure level.

**(d)**

**(c)**

**(b)**

**(a)**

**Figure S23.** The (a) top and (c) side views of Cu(111) model; The (b) top and (d) side views of Cu(111)_30_Pt_1_ model.

**Figure S24.** Computed adsorption energy of the H_2_C(OH)O^−^ intermediate on the two model surfaces

**Figure S25.** Optimization of crystal structures of different intermediates on Cu(111).

**Figure S26.** Photographs of a direct formaldehyde fuel cell connected in series.

**Figure S27.** Durability test under a constant cell voltage

**(b)**

**(a)**

**(d)**

**(c)**

**Figure S28.** Calibration curves for the ^1^H NMR quantification of (a, b) formic acid and (c, d) methanol using DMSO as an internal standard.

Since the Cannizzaro reaction (2HCHO +OH^−^→ CH_3_OH + HCOO^−^ ) produces equivalent amounts of methanol (CH_3_OH) and formic acid (HCOOH), the quantity of HCOOH generated by the Cannizzaro reaction can be deduced from the measured amount of methanol.

The specific method for determining the Faradaic efficiency of formic acid is as follows: the peak area ratios of formic acid and methanol to that of DMSO (internal standard) in the ^1^H NMR spectrum were substituted into the calibration curve to obtain the concentrations of formic acid and methanol in the solution. By subtracting the portion generated from the Cannizzaro reaction from the total formic acid production, the amount of formic acid produced by the formaldehyde oxidation reaction (FOR) was determined. The Faradaic efficiency was then calculated using the formula:

𝐹𝐸 (𝑙𝑖𝑞𝑢𝑖𝑑 − 𝑝ℎ𝑎𝑠𝑒) = 𝛼 × 𝑛 × 𝐹 / 𝑄
